# Nano-Hydroxyapatite from White Seabass Scales as a Bio-Filler in Polylactic Acid Biocomposite: Preparation and Characterization

**DOI:** 10.3390/polym14194158

**Published:** 2022-10-04

**Authors:** Preeyaporn Injorhor, Tatiya Trongsatitkul, Jatuporn Wittayakun, Chaiwat Ruksakulpiwat, Yupaporn Ruksakulpiwat

**Affiliations:** 1 School of Polymer Engineering, Institute of Engineering, Suranaree University of Technology, Nakhon Ratchasima 30000, Thailand; 2 Research Center for Biocomposite Materials for Medical Industry and Agricultural and Food Industry, Nakhon Ratchasima 30000, Thailand; 3 School of Chemistry, Institute of Science, Suranaree University of Technology, Nakhon Ratchasima 30000, Thailand

**Keywords:** fish scales, hydroxyapatite, deproteinization, polylactic acid (PLA), physical properties, mechanical properties, thermal properties, electrospinnability

## Abstract

Nano-hydroxyapatite (nHAp) as a bio-filler used in PLA composites was prepared from fish by acid deproteinization (1DP) and a combination of acid-alkali deproteinization (2DP) followed by alkali heat treatment. Moreover, the PLA/nHAp composite films were developed using solution casting method. The mechanical and thermal properties of the PLA composite films with nHAp from different steps deproteinization and contents were compared. The physical properties analysis confirmed that the nHAp can be prepared from fish scales using both steps deproteinization. 1DP-nHAp showed higher surface area and lower crystallinity than 2DP-nHAp. This gave advantage of 1DP-nHAp for use as filler. PLA composite with 1DP-nHAp gave tensile strength of 66.41 ± 3.63 MPa and Young’s modulus of 2.65 ± 0.05 GPa which were higher than 2DP-nHAp at the same content. The addition of 5 phr 1DP-nHAp into PLA significantly improved the tensile strength and Young’s modulus. PLA composite solution with 1DP-nHAp at 5 phr showed electrospinnability by giving continuous fibers without beads.

## 1. Introduction

Fish waste of more than 7.2 million tons is annually produced and discarded around the world, leading to environmental problems. Fish scale is one of the wastes from the aquaculture sector and fish markets which has not been used much commercially. Fish scales comprise functional materials such as collagen and hydroxyapatite (HAp) and could be the sources of sustainable biomaterials in various applications, especially biomedical applications [1,2,3,4,5,6]. HAp from fish scales is an attractive biomaterial with excellent bioactivity, osteointegration, and osteoconductivity [7,8,9]. It has been used as biosorbent for dyes and metal ions [9]. Therefore, the utilization of fish scales by converting them into high-value materials reduces the waste that causes environmental problems and develops low-cost medical materials. Fish scales comprise HAp and type I collagen [9]. Notably, teleost fish such as sea bass have elasmoid scale, which is similar to the bone composed of extracellular matrix, mainly type I collagen fibers and needle-like hydroxyapatite. Moreover, important anions such as Cl^−^ and F^−^ and cations such as Mg^2+^, Al^3+^, Sr^2+^, Zn^2+^, K^+^, and Na^+^ are presence as trace elements [10,11,12,13,14]. HAp is an interesting type of calcium phosphate with the theoretical chemical formula ${\mathrm{Ca}}_{10}{{(\mathrm{PO}}_{4})}_{6}{\left(\mathrm{OH}\right)}_{2}$. It is used in biomedical applications such as bone replacement material, dental implants, and bone tissue engineering [15,16].

HAp could be prepared or extracted from fish scales by various methods. There are two main methods to eliminate collagen constituents: calcination and alkali heat treatment [3,9,17,18,19,20]. Kongsri et al. [19] prepared nanocrystalline HAp from fish scale waste using the alkali heat treatment method. The method consists of deproteinization by acid and alkali, followed by alkali heat treatment. The products are nanocrystals HAp with high porosity. Although nano-sized HAp (nHAp) can be prepared using alkali heat treatment it requires two steps deproteinization or acid-alkali deproteinization (2DP). Some reports say HAp could be prepared from fish scales by chemical deproteinization [3,8,9] but it takes two times to remove residue protein composition. Hence, this study aims to reduce the steps of deproteinization to obtain nHAp. One-step of deproteination or acid deproteinization (1DP) is used before alkali heat treatment. White seabass is an economically significant species. They are widely cultivated in Thailand. In 2021, white seabass production presented 97.43% of all marine fish farming production. The total commodity value was 5.05 billion baht [21]. Its huge production makes the scales abundant and could be considered a sustainable source of HAp.

Polylactic acid or PLA is a thermoplastic polyester that has advantageous characteristics such as renewability, biocompatibility and inherent biodegradability, ease of preparation, non-toxic nature, and the ability to form fibers. It has been utilized in medical applications extensively [22,23]. However, the disadvantages of PLA such as low thermal stability, hydrophobic nature, and high brittleness, still limit some applications [22,24]. The incorporation of HAp could overcome the hydrophobicity of PLA, improve its poor properties, and stimulate the properties of osteoconduction and osseointegration of the implanted scaffold [7].

This study aims to reduce the step of nHAp preparation and to compare the physical properties of nHAp from 1DP and 2DP. The nHAp samples from 1DP and 2DP with 5 phr are used to form a composite with PLA. The mechanical and thermal properties of both composites are compared. The sample with better performance is further investigated at 2.5, 5, and 10 phr to determine the optimum amount for PLA composite. The PLA/nHAp composite films are fabricated by solvent casting. Effects of deproteinization steps and 1DP-nHAp content on mechanical and thermal properties of the PLA/nHAp composite films are investigated. Moreover, the electrospinnability of PLA/1DP-n${\mathrm{HAp}}_{5}$ is studied. The obtained nHAp is expected to be useful as a bio-filler in PLA for medical applications.

## 2. Materials and Methods

### 2.1. Preparation of nHAp Powder from White Sea Bass Scales

White seabass scales of approximately 10 kg were collected from the local market in Rayong, Thailand, washed with deionized water, and oven-dried at 80 °C. Polylactic acid (PLA, Ingeo™ Biopolymer 4043D-General Purpose Grade) was used as the matrix of composites supplied by NatureWorks Llc. (Minnetonka, MN, USA). Hydrochloric acid (HCl) 37% RPE, sodium hydroxide (NaOH) 99% RPE-ACS, and dichloromethane (DCM) RPE were purchased from Carlo Erba (Milano, Italy).

**Figure 1:**
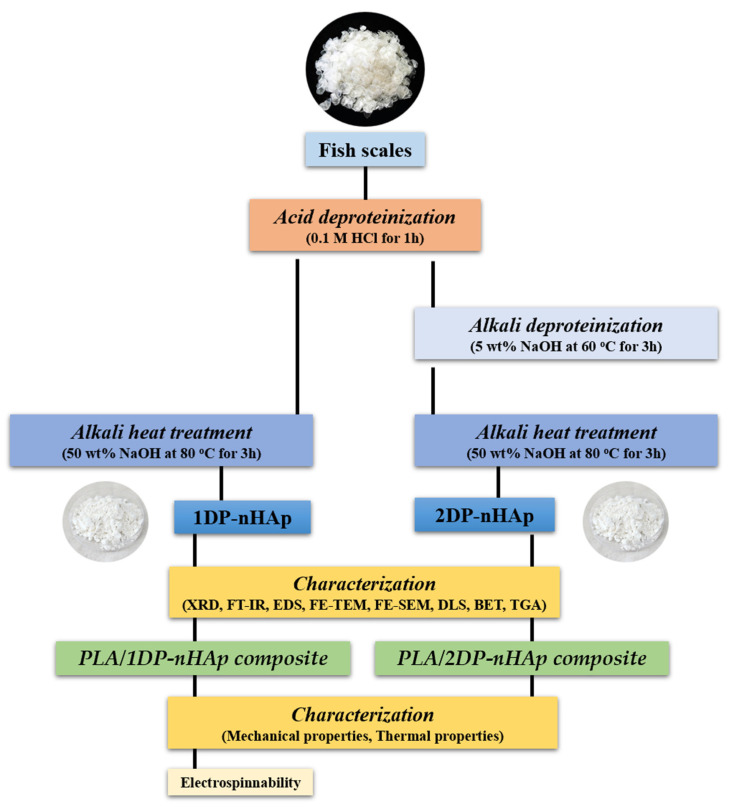
Schematic diagram showing the preparation of nHAp from white seabass scales by alkali heat treatment method.

The preparation method of nHAp was adapted from Kongsri et al. [19]. The steps of preparation are shown in Figure 1. The dried fish scales were treated with 0.1 M HCl for 1 h at room temperature to eliminate collagen, non-collagen proteins, and limiting layers of fish scales. Then, they were washed with deionized water several times before oven-drying at 80 °C. The remaining was treated with 5% (*w*/*v*) NaOH under stirring at 250 rpm for 3 h at 60 °C. The obtained fish scale powder was washed until the pH = 6.5–7 and oven-dried at 80 °C. The alkali heat treatment method was performed on the fish scale powder from the previous step by treating with 50% (*w*/*v*) NaOH under stirring at 250 rpm for 3 h at 80 °C to produce a slurry. The nHAp slurry was washed with deionized water until the pH = 6.5–7 and oven-dried at 80 °C. The final product was nHAp powder. This process is two-steps deproteinization (2DP-nHAp). For one-step of deproteinization (1DP-nHAp), the process was carried out without 5% (*w*/*v*) NaOH treatment.

### 2.2. Preparation of PLA/nHAp Composite Films

To compare nHAp from one-step and two-steps preparation, the powder of 1DP-nHAp and 2DP-nHAp at the same content (5 phr) was dispersed using a magnetic stirrer in DCM for 24 h. To study the effect of nHAp contents, 1DP-nHAp at 2.5 and 10 phr was dispersed with the same procedure. Then, the dispersed nHAp was poured into PLA solution which was dissolved in DCM at a concentration of 10 wt%. The mixture was mixed for 72 h using a magnetic stirrer until a homogenous solution was obtained. The composite films were prepared by casting PLA/nHAp solution into a Petri glass and drying at room temperature for 24 h before evaporating the residue solvent at 40 °C for 72 h in an oven. After that, the PLA/nHAp composite films were stored in a desiccator for further characterization. The effect of nHAp contents on mechanical properties and thermal properties was studied by adding 1DP-nHAp at 2.5, 5, and 10 phr to fabricate PLA/nHAp composite films.

### 2.3. Characterization of nHAp Powder

The crystalline phase and degree of crystallinity ($\chi_{c}$) of nHAp powder were determined by X-ray diffraction (XRD, D2 PHASER, Bruker, Billerica, MA, USA) with Cu Kα radiation source operated at 30 kV and current 10 mA. Bragg’s angle of diffraction (2θ) was measured from 10° to 60° at a scan rate of 2°/min and a step size of 0.02°. The $\chi_{c}$ was calculated by the Equation (1) [25]:

(1) $$\chi_{c}{=(A}_{C}{\;/\;A}_{T})\;\times \;100\%$$

where $A_{C}$ is the area of crystalline peaks and $A_{T}$ is the total area of amorphous and crystalline peaks, respectively.

The crystallite size ($D_{\mathrm{hkl}}$) of the samples was calculated from the Scherrer equation (Equation (2)) [26]:

(2) $$D_{\mathrm{hkl}\;}=\frac{k\lambda }{\beta \cos\theta }$$

where λ is the wavelength of the X-ray radiation, k is the Scherrer constant generally taken to be 0.9, θ is the diffraction angle, and β refers to the full width at half maximum (FWHM).

The functional groups of nHAp powder were analyzed by Fourier transform infrared (FT-IR) spectroscopy on Tensor 27 (Bruker, Billerica, MA, USA) with 64 scans and a resolution of 4 cm**^−^**^1^. Each sample was oven-dried at 110 °C for 24 h, mixed with dried potassium bromide (KBr), mashed in an agate mortar and pressed into a disk. Percentage by weight of carbonate content (wt% ${\mathrm{CO}}_{3}$) of HAp structure was calculated by Equation (3) [27]:

(3) $${\mathrm{wt}\%\;\mathrm{CO}}_{3}=28.62\;\times \;r_{c/p}+0.0843$$

where $r_{c/p}$ is the ratio of the integrated the area between area of $v_{3}$(${\mathrm{CO}}_{3}$) and area of $v_{1}v_{3}$(${\mathrm{PO}}_{4}$) from the absorbance spectra calculated from OriginPro software (OriginPro 2022, OriginLab, Northampton, MA, USA).

The elemental composition of nHAp was analyzed by energy dispersive X-ray spectroscopy (EDS) in a scanning transmission electron microscope (STEM, Talos F200X, Thermo Fisher Scientific, Waltham, MA, USA).

Micrographs of nano-sized HAp from fish scales were acquired using field emission transmission electron microscopy (FE-TEM, Talos F200X, Thermo Fisher Scientific, Waltham, MA, USA). The microstructure of nHAp powder was observed using field emission scanning electron microscopy (FE-SEM, Carl Zeiss Auriga, Oberkochen, Germany) at 3 kV. The samples were sputter-coated with gold for 3 min at 10 mA.

Particle size distribution was analyzed with dynamic light scattering (DLS) using Zetasizer-ZS (Malvern Panalytical, Malvern, UK). The samples were dispersed in ethanol and analyzed at 25 °C. The average particle size in this study was obtained from Z-average.

Nitrogen adsorption–desorption analysis was performed on BelSorp-Mini II (Micro-tracBEL, Osaka, Japan). The nHAp was degassed at 150 °C for 24 h before the analysis. The specific surface area was calculated by Brunauer–Emmett–Teller (BET) method.

Thermal properties, including decomposition temperatures, weight loss, and remaining residue were investigated by thermogravimetric analysis (TGA, TGA/DSC1, Mettler Toledo, Schwerzenbach, Switzerland). Approximately 10 mg of sample was placed on the alumina crucible and heated at a rate of 10 °C/min from 30 to 1000 °C for the analysis under an air atmosphere.

### 2.4. Characterization of PLA/nHAp Composite Films

Tensile properties of PLA/nHAp composite films were measured according to ASTM D882-10 using a universal testing machine (INSTRON/5565, Norwood, MA, USA) with a load cell of 5 kN and a crosshead speed of 250 mm/min at room temperature. The specimens with 1 cm width and 10 cm length were analyzed. Tensile strength, elongation at break, and Young’s modulus of PLA/nHAp composite films were obtained from the average results of five test specimens.

Thermal properties of PLA/nHAp composite films such as enthalpy of melting ($\Delta H_{m}$), enthalpy of cold crystallization ($\Delta H_{\mathrm{cc}}$), glass transition temperature ($T_{g}$), cold crystallization temperature ($T_{\mathrm{cc}}$), melting temperature ($T_{m}$) was evaluated by using differential scanning calorimeter (DSC, Pyris Diamond DSC, Perkin Elmer, Waltham, MA, USA). The samples were characterized under nitrogen flow rate at 20 mL/min from 25 to 200 °C and a heating rate of 10 °C/min. The degree of crystallinity was calculated according to Equation (4) [28]:

(4) $$\chi_{c}=\left[\frac{\left(\Delta H_{m\;}-\Delta H_{\mathrm{cc}}\right)}{\left(\Delta H_{m}^{0}\times \;w\right)}\right]\times 100\%$$

where $\Delta H_{m}^{0}$ is the heat of melting of purely crystalline PLA (93 J·g^−1^), w is the weight fraction of PLA in the sample.

### 2.5. Preparation of PLA/nHAp Fibers by Electrospinning Technique and Their Electrospinnability

The aggregation of nHAp may affect the viscosity of PLA/nHAp composite solution which is one of the factors that affect the electrospinning process. To determine electrospinnability, PLA/1DP-n${\mathrm{HAp}}_{5}$ composite solution was fabricated by electrospinning. Nanofibers were spun at 150 mm distance to a drum collector, which was covered with aluminum foil. The collector rotation speed was set at 300 rpm. The high voltage between the needle tip and the drum collector was set to 25 and 30 kV. The PLA/1DP-n${\mathrm{HAp}}_{5}$ solution was fed at a constant flow rate of 1.0 mL/h. Electrospun fibers were performed by SEM (JSM-6010LV, JOEL, Akishima, Tokyo, Japan) with EDS (EDAX Genesis 2000, AMETEX, Berwyn, PA, USA) to observe their morphology and check the distribution nHAp particles in fibers. The fiber diameter was measured from SEM images using image analysis software (Image J 1.53k, Wayne Rasband and contributors, National Institutes of Health, Bethesda, MD, USA).

## 3. Results and Discussion

### 3.1. Characterization of nHAp Powder

**Figure 2:**
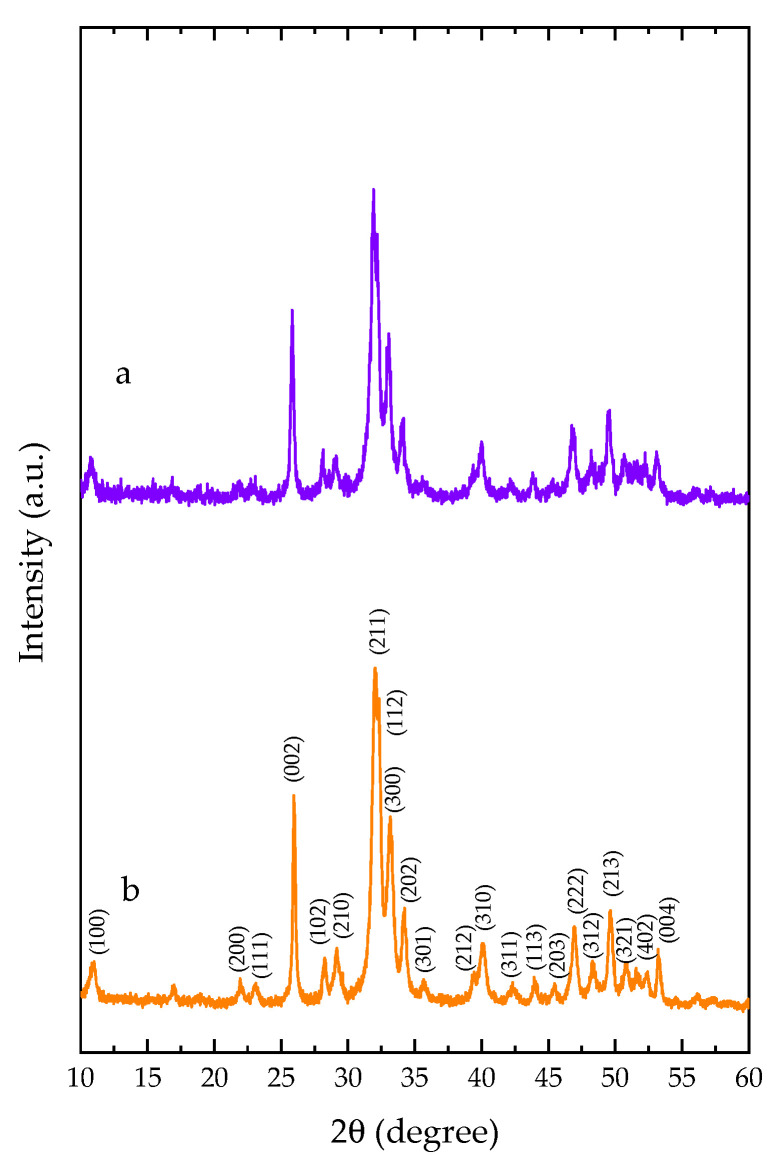
XRD patterns of nHAp powder (a) 1DP-nHAp and (b) 2DP-nHAp.

The XRD patterns of 1DP-nHAp and 2DP-nHAp are shown in Figure 2. The characteristic peaks of nHAp powder are compared with Crystallography Open Database (COD 9003552) for hexagonal HAp structure. The results show that diffraction peaks of both samples correspond to the HAp planes [19,29,30,31]. They only show the crystalline phase of HAp without other phases. All diffraction peaks agree with the standard XRD pattern of hydroxyapatite in Crystallography Open Database (COD 9003552) and the standard of JCPDS card no. 09-0432. Both samples are hexagonal structures with α = β = 90°, and γ = 120°. Corresponding to Sathiskumar et al. [6] nHAp from *Cirrhinus mrigala* fish scales using the same method (2DP) have similar characteristic peaks; this corroborates the method of purity nano-sized HAp preparation. However, their nHAp crystallinity is close to the crystallinity of 1DP-nHAp. It indicated that the deproteinization reduction method could provide nHAp. The crystallinity of 2DP-nHAp is slightly higher than 1DP-nHAp, indicating that 2DP increased the crystallinity of nHAp powder. As a result, 1DP-nHAp with lower crystallinity may be more suitable for enhancing biodegradation behavior and higher metabolic [29]. The degree of crystallinity and crystallite size of nHAp powder are included in Table 1.

**Figure 3:**
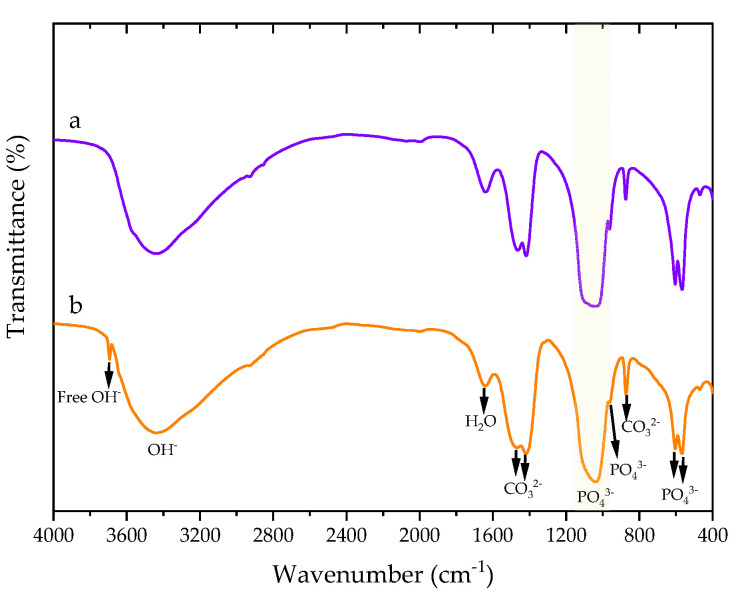
FT-IR transmittance spectra of nHAp powder (a) 1DP-nHAp and (b) 2DP-nHAp.

**Figure 4:**
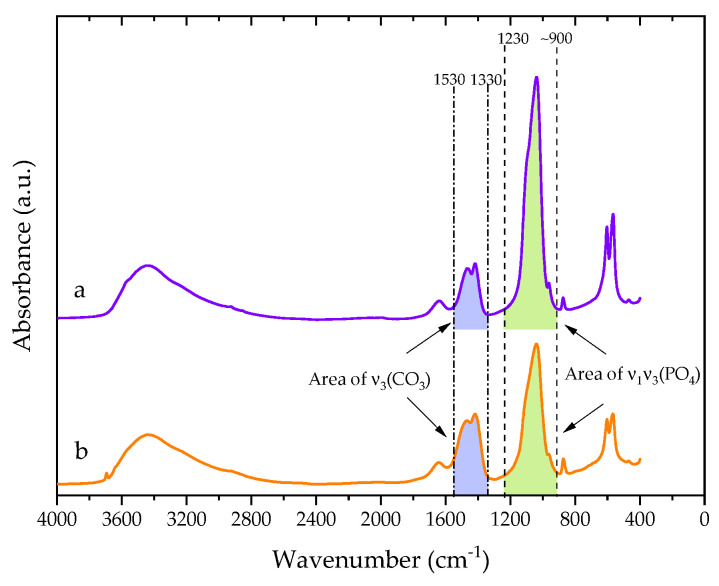
FT-IR absorbance spectra of nHAp powder (a) 1DP-nHAp and (b) 2DP-nHAp.

FT-IR spectra of 1DP-nHAp and 2DP-nHAp in transmission mode are shown in Figure 3. Both samples show a broad peak of associating hydroxyl stretching of adsorbed water around 3500 cm^−1^. The bending mode of the water molecule appears at 1639 cm^−1^. The bands at 1458, 1417, and 874 cm^−1^ are assigned to carbonate groups in nHAp powder. It indicated the nHAp powder is B-type carbonated hydroxyapatite with carbonate ions substituting phosphate ions in the hydroxyapatite structure. The strong broadband between 1083–1042 cm^−1^ and the bands at 962, 603, and 565 cm^−1^ correspond to phosphate groups [8,32,33]. The percent weight of carbonate in 1DP-nHAp and 2DP-nHAp, calculated from the band in absorption mode as shown in Figure 4, are 6.57 and 15.64 wt%, respectively. It suggested the presence of carbonate ions substituting phosphate ions more than 1DP-nHAp. In addition, the spectrum of 2DP-nHAp showed the stretching of free hydroxyl groups at 3642 cm^−1^. It is in agreement with the report by Gergely et al. [34] that the free hydroxyl groups may connect to calcium oxide on the surface. In addition, The FT-IR results of 1DP-nHAp and 2DP-nHAp are similar to Gopalu et al. [35]. They presented FT-IR and Raman results of pure HAp. It can be assumed that 1DP-nHAp and 2DP-nHAp have the same characteristics as pure HAp.

**Figure 5:**
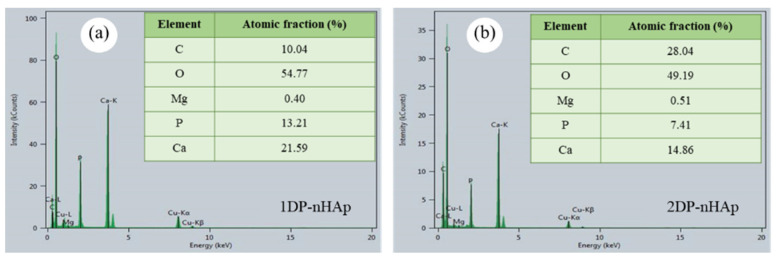
Results from elemental analysis by EDS of nHAp powder (**a**) 1DP-nHAp and (**b**) 2DP-nHAp.

EDS spectra of the nHAp with element composition are shown in Figure 5. Both 1DP-nHAp and 2DP-nHAp have constituents carbon (C), oxygen (O), magnesium (Mg), calcium (Ca), and phosphorous (P). Typically, the presence of Mg constituent is a significant factor in bone and teeth growth [8]. Hydroxyapatite is a type of calcium phosphate ceramic which is classified by calcium/phosphorus atomic ratio (Ca/P); for example, hydroxyapatite ${\mathrm{Ca}}_{10}{{(\mathrm{PO}}_{4})}_{6}{\left(\mathrm{OH}\right)}_{2}$, HAp, Ca/P = 1.667) and β-tricalcium phosphate (β-${\mathrm{Ca}}_{3}{{(\mathrm{PO}}_{4})}_{2}$, β-TCP, Ca/P = 1.5) [36]. The Ca/P has illustrated the molar ratio from nHAp. The Ca/P of 1DP-nHAp and 2DP-nHAp are 1.63 and 2.01, respectively. The Ca/P of 1DP-nHAp is close to the theoretical value (1.67) [8]. The Ca/P ratio of 2DP-nHAp is higher than the theoretical value due to the substitution of phosphate ions with carbonate ions. These results are the same as the report by Deb and Deoghare [29] and consistent with the FT-IR results.

**Figure 6:**
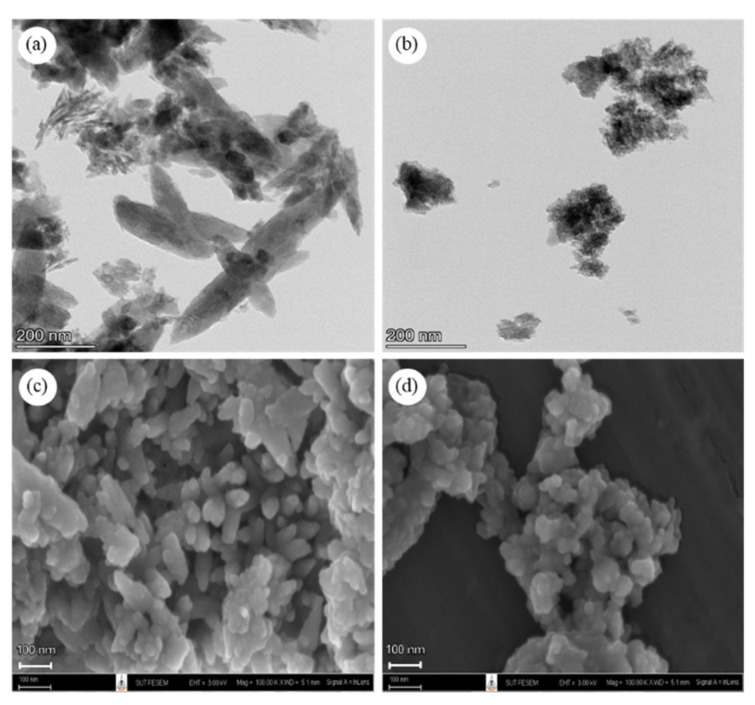
FE-TEM images of (**a**) 1DP-nHAp and (**b**) 2DP-nHAp and FE-SEM images of (**c**) 1DP-nHAp and (**d**) 2DP-nHAp.

The nano-sized particles of nHAp from 1DP-nHAp and 2DP-nHAp were confirmed using FE-TEM, as shown in Figure 6a,b. The particle sizes of both samples in the range of nano-scale. The microstructure is observable in the FE-SEM images. FE-SEM in Figure 6. 1DP-nHAp has rod-like shapes with different widths and lengths (Figure 6c), while 2DP-nHAp has an irregular shape (Figure 6d). Typically, the external elasmodine layer of fish scales is composed of needle-like HAp crystals hybridized with randomly arranged collagen fibers. However, the HAp products from fish scales have various shapes, such as irregular, rod-like, spherical, and needle-like. According to Qin et al. [9], the rod-like shape or needle-like shape of HAp is frequently seen from HAp extracted from natural sources. However, the extraction method or the source has no effect on the shape of HAp particles. The same extraction method can provide a different HAp shape. It can be concluded that 1DP-HAp has the natural shape that is frequently found. Moreover, 2DP-nHAp shows an aggregation of particles that leads to non-homogenous distribution in the matrix and deteriorates mechanical properties [37].

**Figure 7:**
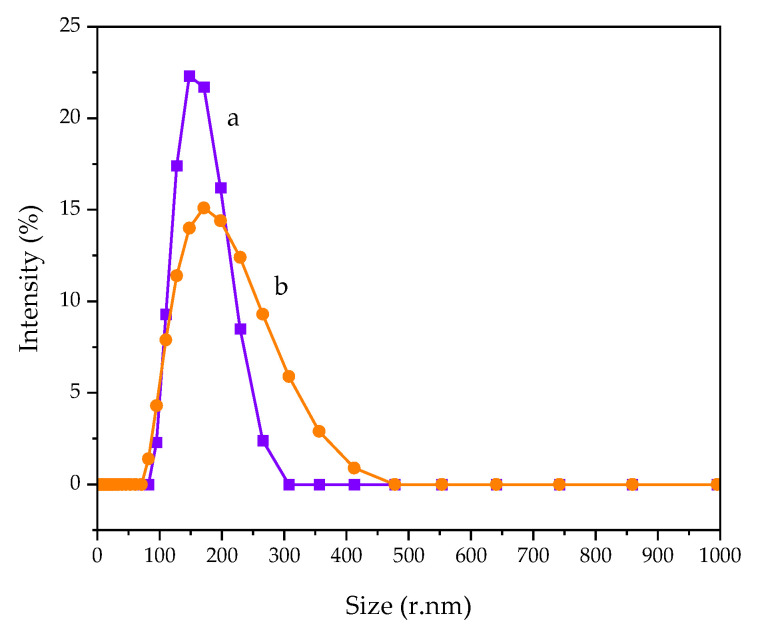
DLS measurements of particle size distribution of nHAp powder (a) 1DP-nHAp and (b) 2DP-nHAp.

Particle size distribution of 1DP-nHAp and 2DP-nHAp measured by DLS technique is shown in Figure 7. The particle size was reported by Z-average particle size, which is the intensity weighted harmonic mean size. The particle size distribution of both samples is a mono-modal distribution with the Z-average particle size of 223.6 and 172.9 nm, respectively. 1DP-nHAp shows a narrower distribution than 2DP-nHAp. According to Raita et al. [38], the size from the DLS technique is larger than that observed from the electron microscopy because DLS measures a hydrodynamic size, rather than a physical one.

**Figure 8:**
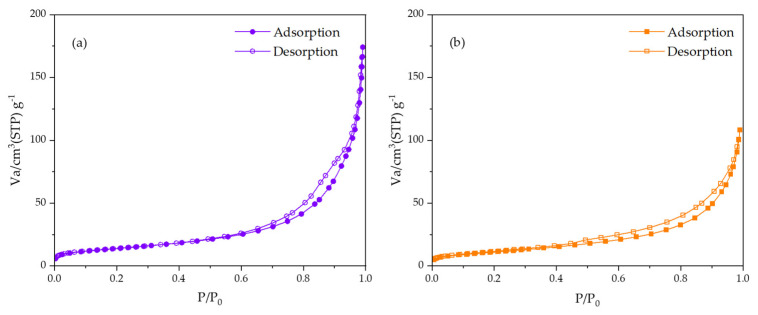
Nitrogen adsorption–desorption isotherms of nHAp powder (**a**) 1DP-nHAp and (**b**) 2DP-nHAp.

The adsorption–desorption isotherms of 1DP-nHAp and 2DP-nHAp are shown in Figure 8. Both samples demonstrate reversible Type II isotherms which are the physisorption on nonporous adsorbents according to the IUPAC classification. Moreover, their adsorption and desorption lines do not overlap, forming a type H3 hysteresis loop which is found on materials with non-rigid aggregates of plate-like particles [39,40]. Their BET surface area, total pore volume, and mean pore diameter are included in Table 1. 1DP-nHAp has a larger surface area than 2DP-nHAp. For tissue engineering, 1DP-nHAp, the sample with a larger surface area, could interact better with osteoblast cells to promote cell growth and proliferation [1,9].

**Figure 9:**
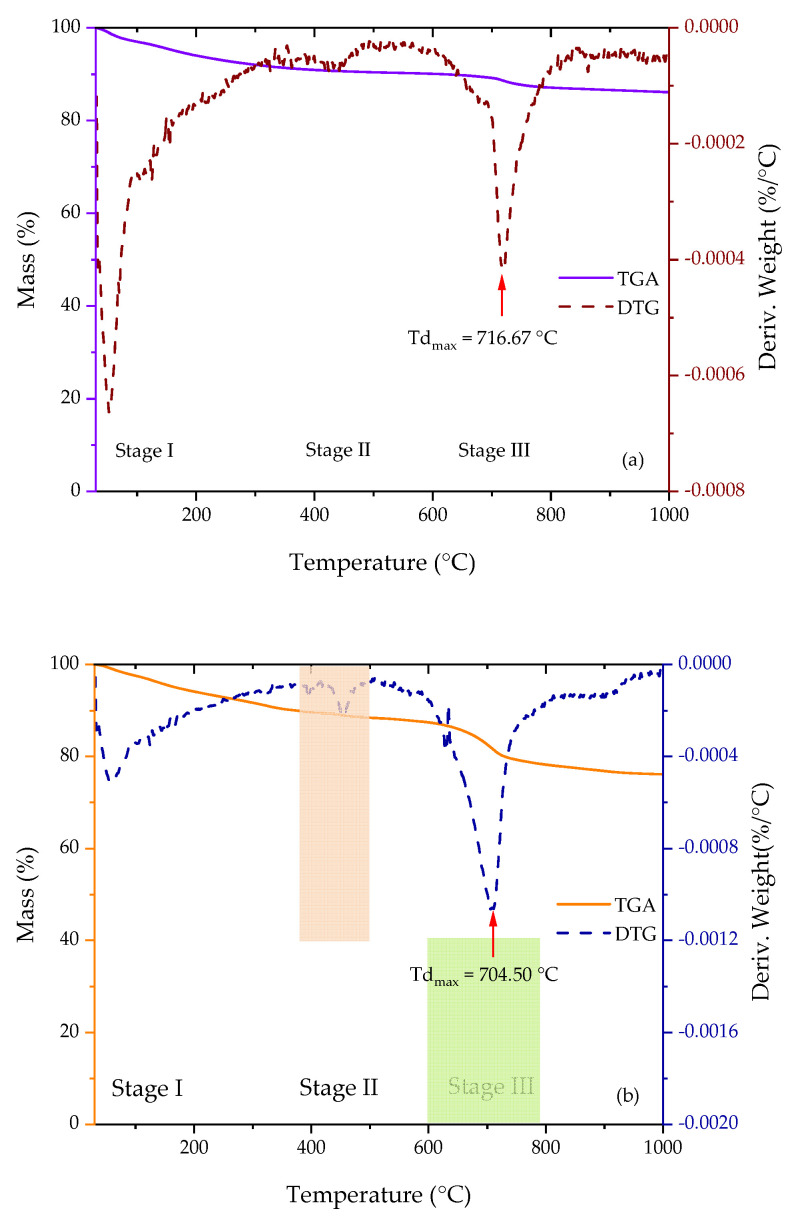
TGA and DTG curves of thermal decomposition of (**a**) 1DP-nHAp and (**b**) 2DP-nHAp.

TGA analysis was used to confirm the composition of HAp in 1DP-nHAp and 2DP-nHAp. Figure 9a,b exhibits the TGA results showing the weight loss of 1DP-nHAp and 2DP-nHAp in the temperature range from 30–1000 °C, respectively. The figure also includes their DTG curves, the derivative of the weight loss, which indicate three stages of weight loss from both samples. The first stage at 30–200 °C is the weight loss from water evaporation. The second stage around 375–500 °C corresponds to the weight loss from the combustion of hydrocarbons which are the organic residue. The final stage, at around 600–800 °C corresponds to the loss of carbonate groups in the nHAp structure [19,41]. According to the FT-IR results, 2DP-nHAp has carbonate groups more than 1DP-nHAp. So, the carbonate weight loss of 2DP-nHAp is greater. The HAp residue of 1DP-nHAp and 2DP-nHAp were 86.10 and 76.12 wt%, respectively. In addition, maximum degradation temperature (${\mathrm{Td}}_{\max}$) of 1DP-nHAp and 2DP-nHAp were 716.67 °C and 704.50 °C, respectively. It indicated that 1DP-nHAp has better thermal stability than 2DP-nHAp.

### 3.2. Characterization of PLA/nHAp Composite Films

**Figure 10:**
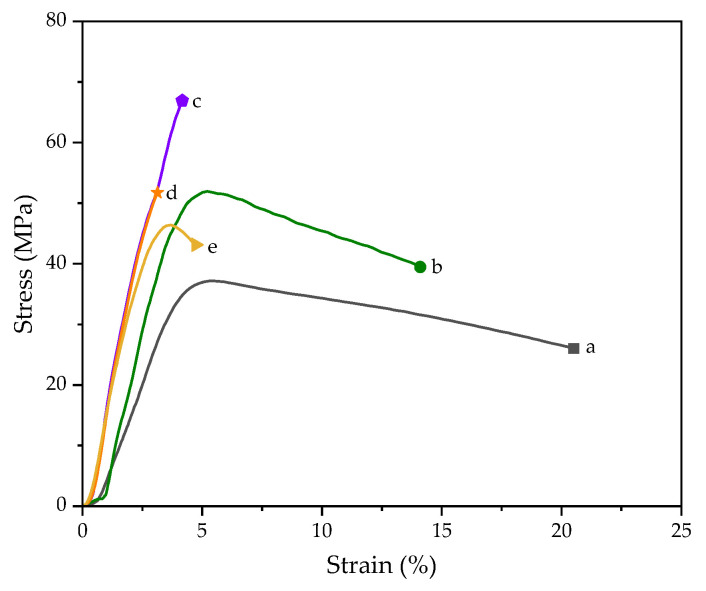
Tensile stress–strain curve of PLA/nHAp composite films with nHAp from different preparation steps and 1DP-nHAp at various contents (a) PLA, (b) PLA/1DP-n${\mathrm{HAp}}_{2.5}$, (c) PLA/1DP-n${\mathrm{HAp}}_{5}$, (d) PLA/2DP-n${\mathrm{HAp}}_{5}$, and (e) PLA/1DP-n${\mathrm{HAp}}_{10}$.

The PLA/nHAp composite films were fabricated by the solution casting method. 5phr of 1DP-nHAp and 2DP-nHAp was added for comparison. Figure 10 shows the tensile stress–strain curve of the PLA/nHAp composite films with nHAp from different preparation steps and with different 1DP-nHAp contents. The results of the tensile properties are shown in Table 2. It shows Young’s modulus, tensile strength, and elongation at break of PLA and PLA/nHAp composite films. The Young’s modulus of PLA was 1.73 ± 0.18 GPa and the value for the PLA/1DP-n${\mathrm{HAp}}_{5}$ and PLA/2DP-n${\mathrm{HAp}}_{5}$ increased up to 2.65 ± 0.05 MPa and 2.38 ± 0.11 MPa, respectively. However, elongation at break of PLA/2DP-n${\mathrm{HAp}}_{5}$ is lower than PLA/1DP-n${\mathrm{HAp}}_{5}.\;$According to Kamarudin et al. [42] and Boey et al. [43], the modulus and strength of the composite depend on mechanical interlocking or chemical interaction between the filler and the matrix. Thus, adhesion or bonding between the filler and the matrix is an important factor. Mechanical interlocking is a form of physical force that holds filler and matrix together, whereas chemical interaction is the formation of chemical bonding via functional groups between filler and matrix. In this work, the surface area of nHAp that is available for the mechanical interlocking and the chemical bonding between carbonyl (−COO) of PLA and Ca*^2+^* ions on the surface of nHAp were considered to affect the mechanical properties of the PLA composite [44,45]. The composite with 1DP-n${\mathrm{HAp}}_{5}$ addition showed the highest tensile strength (66.41 ± 3.63 MPa). This result corresponds to the surface properties of 1DP-nHAp which has larger surface area and could cause more mechanical interlocking with matrix than 2DP-nHAp. So, the interlocking between filler and matrix was expected that greater. According to EDS results, 1DP-nHAp has atomic fraction of Ca*^2+^* more than 2DP-nHAp, indicating that 1DP-nHAp has more interaction sites. This assumption supplemented that the strength of PLA/1DP-n${\mathrm{HAp}}_{5}$ could occur from the chemical bonding of −COO and Ca*^2+^*. However, both samples have mechanical properties which correspond to the tensile strength of human skeletal bones (ranging from 40*–*200 MPa) and the critical mechanical modulus of bone replacement material in non-load bearing sites (ranging from 10*–*1500 MPa) [46].

The mechanical properties of the PLA/nHAp composite films with various 1DP-nHAp content are also given in Table 2. The results indicated that PLA/nHAp composite films show more tensile strength than neat PLA films. The tensile strength of PLA/nHAp composite films increases with increasing 1DP-nHAp content up to 5 phr as well as the result of Young’s modulus. It indicated good dispersion of nHAp into PLA matrix and strong interfacial actions between the PLA and nHAp [46]. Therefore, the optimum content of nHAp is 5phr. However, elongation at break is the lowest. Tensile strength decreased with adding nHAp content at 10 phr. The high content of nHAp would agglomerate, causing poor dispersion of nHAp in the PLA matrix leading to the deterioration of tensile strength as previously reported by Li et al. [47]. According to Boey et al. [43], the mechanical properties of composites are found to improve linearly with increasing filler content up to a certain optimum value. Moreover, the addition of filler above that limit adversely affects the mechanical strength due to the formation of agglomerates. Elongation at break of PLA/nHAp composite films decreased by the addition of nHAp up to 5 phr. The results are suggested that the optimum addition of nHAp into the PLA matrix can be improved the rigidity of the composite film. Nevertheless, all composite samples have enough strength for developing medical materials that can be degraded. This strength is an outgrowth from inorganic filler that the materials should maintain sufficient strength while providing specific cell-surface receptors during the tissue remodeling process [48].

**Figure 11:**
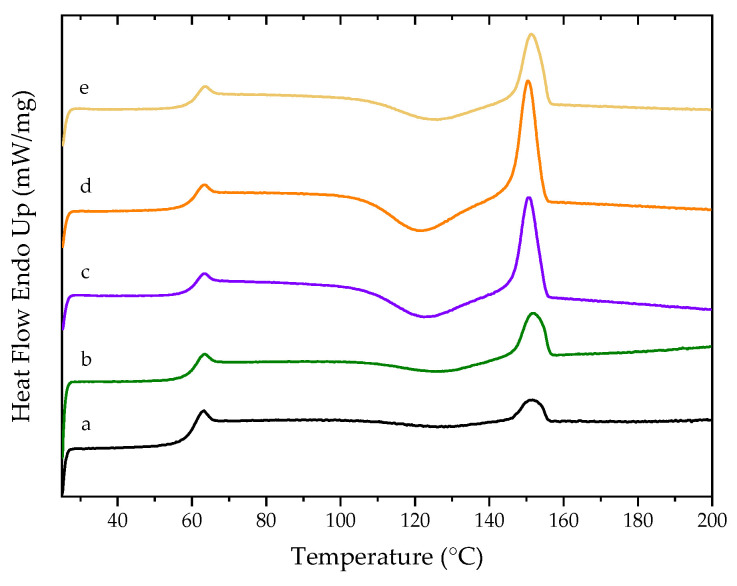
The second heating DSC thermograms of PLA and PLA/nHAp composite films with nHAp from different preparation steps and 1DP-nHAp at various contents (a) PLA, (b) PLA/1DP-n${\mathrm{HAp}}_{2.5}$, (c) PLA/1DP-n${\mathrm{HAp}}_{5}$, (d) PLA/2DP-n${\mathrm{HAp}}_{5}$, and (e) PLA/1DP-n${\mathrm{HAp}}_{10}$.

The thermal properties of PLA and PLA/nHAp composite films was studied by differential scanning calorimetry (DSC). Their DSC thermograms are shown in Figure 11. The effect of the nHAp from different preparation steps on the thermal properties of PLA/nHAp composite films is shown in Table 3. The glass transition temperature ($T_{g}$) of all samples was slightly different, all in the range of 60–61 °C. It indicates that the interaction between matrix and filler is low, and it can slightly change the mobility of polymer chains related to the glass transition. An exothermic peak corresponds to the crystallinity of the PLA. The addition of nHAp particles in the PLA matrix affects the temperature of cold crystallization ($T_{\mathrm{cc}}$) values tend to decrease due to the nHAp particles acting as nucleation centers for PLA crystals [49]. So, the PLA crystallinity was enhanced by loading nHAp due to the exothermic peak, as observed in PLA/nHAp composites being sharper than neat PLA and the inorganic filler could promote the polymer crystallization on their surface [50]. As compared between 1DP-nHAp and 2DP-nHAp at the same filler contents, the $T_{g},{\;T}_{\mathrm{cc}}$, and $T_{m}$ of both composites seem slightly different. Still, their degree of crystallinity is dramatically different because the 2DP-nHAp has smaller particles. The smaller size of 2DP-HAp can generate more cross-linking points in the PLA matrix than 1DP-nHAp, which restricts the movement of the PLA chain [51].

The melting temperature peak of PLA is 151.60 °C while the peak of PLA/nHAp composite films is decreased to a lower temperature. However, it is the same as the neatPLA. Meanwhile, the effect of the 1DP-nHAp content on the thermal properties of PLA/nHAp composite films is shown in Table 3. The $T_{g}$ of PLA was 60.10 °C, while $T_{g}$ of the PLA/nHAp composites slightly increased with increasing 1DP-nHAp content. This change indicated that the interactions of the 1DP-nHAp slightly interfere with the mobility of polymer chains related to the glass transition [52]. The $T_{\mathrm{cc}}$ value of the PLA and PLA/nHAp composites are 127.89, 128.39, 122.40, and 126.07 °C when 0, 2.5, 5, and 10 phr of 1DP-nHAp were added, respectively. It indicated that the 1DP-nHAp at 5 and 10 phr accelerated the cold crystallization of PLA due to the ability of 1DP-nHAp to induce heterogeneous nucleation into the PLA matrix. However, adding 1DP-nHAp enhanced the PLA’s crystallinity compared with neat PLA. The crystallinity corresponds to $T_{m}$; typically, the polymers melt at a higher temperature when they form fewer perfect crystals [53,54].

**Figure 12:**
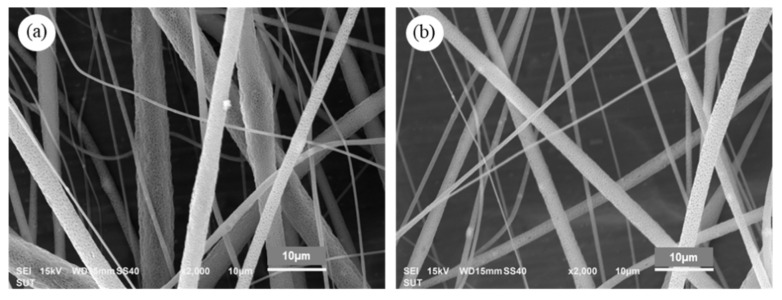
SEM images of PLA/1DP-n${\mathrm{HAp}}_{5}$ composite fibers operated different applied high voltage (**a**) 25 kV and (**b**) 30 kV.

SEM images of PLA/1DP-n${\mathrm{HAp}}_{5}$ composite fibers with applied high voltage at 25 and 30 kV are shown in Figure 12. The continuous PLA/1DP-n${\mathrm{HAp}}_{5}$ composite fibers with rough surfaces without the formation of beads were successfully fabricated. The average diameter of the fibers obtained from the high voltage of 25 and 30 kV is 3.83 ± 1.09 and 2.62 ± 0.35 µm, respectively. The fiber diameter from 30 kV high voltage shows a slightly smaller fiber diameter than that from 25 kV. The increase in the applied voltage leads to the stretching of the polymer chains in correlation with the charge repulsion within the polymer jet [55].

**Figure 13:**
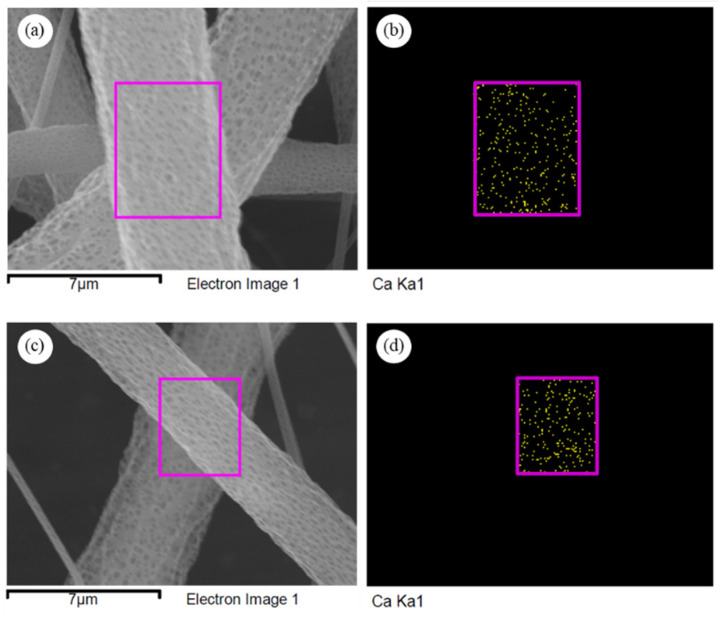
EDS analyzed area and EDS mappings of PLA/1DP-n${\mathrm{HAp}}_{5}$ composite fibers operated different applied high voltage (**a**,**b**) 25 kV and (**c**,**d**) 30 kV.

EDS analyzed area and mappings of PLA/1DP-n${\mathrm{HAp}}_{5}$ at 25 and 30 kV high voltage are shown in Figure 13. The EDS analyzed area of each sample was shown by pink frame. The existence of calcium was indicated to nHAp that disperse in fibers. The EDS mappings show a good dispersion of nHAp in both samples. This indicated that nHAp could be incorporated into PLA solution without phase separation and aggregation.

## 4. Conclusions

Nano-hydroxyapatite (nHAp) was prepared from white seabass scales by two methods: (1) acid deprotonization (1DP) and (2) combination of acid-alkali deproteinization (2DP) followed by alkali heat treatment. Physicochemical properties of 1DP-nHAp and 2DP-nHAp are compared by several techniques. Both samples are B-type carbonated hydroxyapatite. Their particles are non-porous with irregular shapes, and nano-sized diameters. 1DP-nHAp has lower crystallinity, narrower particle size distribution, lower Ca/P ratio and larger surface area. 1DP-nHAp and 2DP-nHAp are used as bio-fillers for polylactic acid (PLA). The composite films PLA/1DP-nHAp and PLA/2DP-nHAp are compared in terms of mechanical strength and thermal behavior. PLA/1DP-n${\mathrm{HAp}}_{5}$ is the better composite. Both composites have higher Young’s modulus and tensile strength than the neat PLA but shorter elongation at break. At the same nHAp content (5 phr), the composite film PLA/1DP-n${\mathrm{HAp}}_{5}$ shows higher Young’s modulus and tensile strength with higher elongation at break than the composite film PLA/2DP-n${\mathrm{HAp}}_{5}$. The strength of PLA/1DP-nHAp decreases by increasing or decreasing the nHAp content. The thermal behaviors of all PLA/nHAp composite films are slightly different from the neat PLA. The interaction between matrix and filler is low, and it slightly changes the mobility of polymer chains. The nHAp can induce heterogeneous nucleation into the PLA matrix via accelerated cold crystallization. Moreover, ${\mathrm{PLA}/1\mathrm{DP}-\mathrm{nHAp}}_{5}$ demonstrates good eletrospinnability, producing continuous fibers without beads. The nHAp dispersed well in PLA without phase separation and aggregation.

## Figures and Tables

**Table 1:** The analysis information of n-HAp powder.

Properties	Materials	
	1DP-nHAp	2DP-nHAp
Crystallinity (%$\chi_{c}$)	71.41	80.99
Crystallite size, $D_{\mathrm{hkl}}$(nm)	19.41	13.87
BET surface area (cm^3^/g)	50	41
Total pore volume (cm^3^/g)	0.26	0.17
Mean pore diameter (nm)	21.25	16.32

**Table 2:** Mechanical properties of PLA/nHAp composite films with nHAp from different steps of preparation and 1DP-nHAp at various contents.

Designation	nHAp Content (phr)	Young’s Modulus (GPa)	Tensile Strength (MPa)	Elongation at Break (%)
PLA	−	1.73 ± 0.18	38.21 ± 0.95	23.39 ± 1.97
$\mathrm{PLA}/1\mathrm{DP}-n{\mathrm{HAp}}_{2.5}$	2.5	1.94 ± 0.27	54.45 ± 1.42	14.74 ± 2.92
$\mathrm{PLA}/1\mathrm{DP}-n{\mathrm{HAp}}_{5}$	5	2.65 ± 0.05	66.41 ± 3.63	4.32 ± 0.34
$\mathrm{PLA}/2\mathrm{DP}-n{\mathrm{HAp}}_{5}$	5	2.38 ± 0.11	52.21 ± 4.67	3.44 ± 0.66
$\mathrm{PLA}/1\mathrm{DP}-n{\mathrm{HAp}}_{10}$	10	2.02 ± 0.18	45.80 ± 1.78	4.72 ± 0.59

**Table 3:** Thermal characteristics of PLA and PLA/nHAp composite films with nHAp from different preparation steps and 1DP-nHAp at various contents.

Designation	T_g_ (°C)	T_cc_ (°C)	∆H_cc_ (J·g^−1^)	T_m_ (°C)	∆H_m_ (J·g^−1^)	X_c_ (%)
PLA	60.10	127.89	3.57	151.60	3.16	7.23
$\mathrm{PLA}/1\mathrm{DP}-n{\mathrm{HAp}}_{2.5}$	60.81	128.39	6.71	151.59	6.51	14.50
$\mathrm{PLA}/1\mathrm{DP}-n{\mathrm{HAp}}_{5}$	61.00	122.40	12.98	150.61	15.69	32.38
$\mathrm{PLA}/2\mathrm{DP}-n{\mathrm{HAp}}_{5}$	60.77	121.90	19.72	150.44	16.74	41.17
$\mathrm{PLA}/1\mathrm{DP}-n{\mathrm{HAp}}_{10}$	61.14	126.07	11.22	151.28	10.21	25.35

## Data Availability

Not applicable.
